# The Comparison of High-Intensity Interval Training Versus Moderate-Intensity Continuous Training after Coronary Artery Bypass Graft: A Systematic Review of Recent Studies

**DOI:** 10.3390/jcdd9100328

**Published:** 2022-09-28

**Authors:** Billie Schulté, Lisa Nieborak, Franck Leclercq, Jorge Hugo Villafañe, Eleuterio A. Sánchez Romero, Camilo Corbellini

**Affiliations:** 1 Department of Physiotherapy, LUNEX International University of Health, Exercise and Sports, 50, Avenue du Parc des Sports, 4671 Differdange, Luxembourg; 2 IRCCS Fondazione Don Carlo Gnocchi, Piazzale Morandi 6, 20148 Milan, Italy; 3 Musculoskeletal Pain and Motor Control Research Group, Faculty of Sport Sciences, Universidad Europea de Madrid, 28670 Madrid, Spain; 4 Musculoskeletal Pain and Motor Control Research Group, Faculty of Health Sciences, Universidad Europea de Canarias, 38300 Tenerife, Canary Islands, Spain; 5 Department of Physiotherapy, Faculty of Sport Sciences, Universidad Europea de Madrid, 28670 Villaviciosa de Odón, Madrid, Spain; 6 Department of Physiotherapy, Faculty of Health Sciences, Universidad Europea de Canarias, 38300 Tenerife, Canary Islands, Spain

**Keywords:** cardiac rehabilitation, high-intensity interval training, quality of life, coronary artery bypass

## Abstract

Currently, no international consensus on cardiac rehabilitation exists, leading to great variability in the intensity recommendations for training programs for cardiac patients, including those undergoing coronary artery bypass graft surgery (CABG). While some countries prefer the high-intensity interval training (HIIT) method to improve cardiorespiratory fitness, other countries opt for moderate-intensity continuous training (MICT). The aim of this systematic review was to compare the effects of HIIT and MICT on aerobic fitness and quality of life (QoL) in patients undergoing CABG with the intention of providing support for a consensus on exercise therapy. Methods: A systematic review of randomized controlled trials (RCTs) was conducted using the online publication databases PubMed, the Cochrane Library and the Bibliothèque nationale du Luxembourg (BnL) covering the last ten years to July 2022. Relevant identified studies respecting the inclusion/exclusion criteria were selected, screened and extracted by four reviewers. Furthermore, the methodological quality of the clinical trials was assessed using the PEDro scale, which was reinforced using the Cochrane Risk of Bias Tool for Randomized Trials (RoB2) for the evaluation of the risk of bias to provide more detail in the evaluation. The certainty of the evidence analysis was established using different levels of evidence in accordance with the Grading of Recommendations, Assessment, Development and Evaluation (GRADE) framework. Results: A total of 379 patients from five RCTs diagnosed with coronary artery disease, including patients undergoing CABG, performed aerobic exercise over different time periods and were assessed based on peakVO2, VO_2_max and QoL. Overall, both training methods provided improvements in cardiorespiratory fitness and quality of life, with greater changes in HIIT groups. Conclusion: Both trainings methods provide improvements in cardiorespiratory fitness and QoL, with greater increases from HIIT. The moderate quality of evidence supports the use of HIIT and MICT to improve cardiorespiratory fitness and QoL.

## 1. Introduction

The World Health Organization (WHO) defines cardiac rehabilitation (CR) as a program aiming to improve the physical, mental and social conditions of people suffering from heart diseases [1]. It represents a complex intervention including various components such as health education, counselling on cardiovascular risk decrease, physical activity and stress management [2] and constitutes a fundamental element for heart patients. Different guidelines, namely the National Institute for Health and Care Excellence (NICE), the department of health and the British Association for Cardiovascular Prevention and Rehabilitation (BACPR), established a list of groups benefitting from CR. Coronary artery bypass grafting (CABG) represents one of them and is, with almost 400,000 surgeries each year, the most performed major surgical procedure worldwide [3].

According to Bhatnagar et al. [4], cardiovascular disease (CVD) represents the leading cause of death and constitutes worldwide the principal predictor of disability by the year 2020 [5]. Stating the statistics, CVD is responsible for one-tenth of deaths in patients younger than 35 years, one-third of mortalities in people aged between 35–45 years and almost three-quarters of deaths in individuals over 45 years [5]. Coronary artery diseases (CAD) are inadequate to be managed with medical treatment and require surgical intervention and, more precisely, CABG. CABG aims to restore blood flow to the ischemic myocardium by bypassing atheromatous blockages in coronary arteries with harvested venous or arterial conduits and provides function restoration, viability and anginal symptom relief [3]. According to Melly et al. [6], the average CABG incidence rate in western European countries is 62 per 100,000 inhabitants, and the prevalence of CAD is constantly increasing [7].

Post CABG surgery, patients are enrolled in CR programs, in which exercise training constitutes a central element and provides positive outcomes [8]. However, despite the popularity of aerobic exercise in CR, the best way to structure the training in terms of mode, frequency, and intensity is still unknown. Furthermore, Taylor et al. [9] claim that no international consensus for CR exists and emphasise the significant variability regarding intensity recommendation between various countries. While Japan and Australia provide light-moderate intensity training, the CR program in the UK or France relies on moderate or even moderate-vigorous intensity as in other European countries [9]. 

HIIT gained popularity in 1950. The repetition of specific short characterises HIIT as prolonged intervals of high-intensity exercise followed by low-intensity exercising or resting active recovery phase [10]. MICT is, according to Williams et al. [11], 30 to 60 min of aerobic exercise training at a peak heart rate of around 64–76%. Aerobic exercise, whether HIIT or MICT, represents a fundamental element in the CR after cardiac surgery and focuses on improving aerobic fitness and functional capacity by using a treadmill, stationary cycling or even walking as main exercises [12]. 

Stating Karampreet [13], The WHO describes maximal oxygen consumption (VO_2_max) as the best and most ubiquitous indicator of cardiorespiratory fitness and constitutes one of the outcome criteria for the present research. VO_2_max is the maximum capacity to uptake, transport and utilise oxygen during intense maximal exercise without changing despite workload increases over time [14]. 

The Peak Oxygen uptake (peakVO_2_), which is directly reflective of the VO_2_max and the highest attained VO_2_ value upon an incremental exercise, represents another established cardiorespiratory fitness measure [15] and is used as an additional outcome in the present systematic review. In case of disability to perform cardiopulmonary exercise testing for diverse reasons, peakVO_2_ can be predicted by applying a generalised equation related to the 6 min walking test (6MWT) [16]. Latter one represents a scientific recognised, simple, and widely used assessment tool in cardiopulmonary rehabilitation and completes the evaluation tests regarding aerobic fitness. 

CABG represents one of the most common cardiac surgeries worldwide and goes along with a significant decrease in QoL and cardiorespiratory fitness [7,16]. Therefore, it is crucial and inevitable to provide the best possible CR, for which, until today, no standard and international consensus exist. Due to the lack of an international consensus on CR and the enormous variability regarding the intensity recommendation, further research to provide optimal knowledge concerning the training intensity in CR would greatly enrich the rehabilitation process across the different phases of aerobic exercise training in CR. 

This systematic review aims to compare high-intensity interval training (HIIT) versus traditional moderate-intensity continuous training (MICT) in patients undergoing CABG surgery concerning VO_2_max, peakVO_2_, 6MWT and QoL. 

## 2. Materials and Methods

The following study design represents a systematic review including collected and analysed analytical data from different databases and is based on considering the Preferred Reporting Items for Systematic-reviews and Meta-analyses (PRISMA) [17]. Furthermore, the protocol was registered in the International Prospective Register of systematic reviews (PROSPERO) under the following registration number: CRD42022340012.

### 2.1. The PICO Question 

The research question was guided by the PICO framework (patient/intervention/comparison/outcomes) to formulate a well-focused research question to improve the efficiency of literature searching [18]. The present systematic review selected a Population who underwent CABG surgery and performed exercise training as a part of the cardiac rehabilitation program. The therapy comparisons regarded the training strategies: HIIT and MICT (considered the standard therapy intervention). The outcomes measure VO_2_max, peakVO_2_, VO_2_, 6MWT and QoL were identified and described to complete the research question. 

### 2.2. Eligibility Criteria

RCTs and Meta-analyses no older than ten years were included in the systematic review if they met the inclusion criteria: Humans older than 18 years who underwent CABG and performed HIIT or MICT to assess aerobic capacity and QoL. Outcome measures, and more precisely, the VO_2_ max, peakVO_2_, 6MWT and QoL assessments, constituted further eligibility criteria and were considered regarding the study selection. All articles in English were recruited and retained.

Essential exclusion criteria represent people younger than 18 years and chronic patients. Furthermore, studies with assessment outcomes other than those mentioned above and in other languages or periods were eliminated from the selection. Finally, literature reporting only patients undergoing different medical and/or surgical approaches other than CABG or an inappropriate intervention was excluded.

### 2.3. Outcome Measures

Since the present work is a systematic review, no differentiation between primary and secondary outcomes is necessary. The following outcomes have been selected to assess the effect of HIIT or MICT on aerobic capacity: VO_2_ max, peakVO_2_ and 6MWT. Furthermore, the impact of exercise training on QoL represents another outcome measure in the present literature review. 

The VO_2_max, peakVO_2_ and the 6 MWT, an inexpensive, quick, safe and well-tolerated test, act as further scientific recognised outcome measures related to aerobic fitness.

### 2.4. Data Sources and Search Strategy

The present systematic review is based on systematic electronic literature research and includes evidence from different databases, namely PubMed, the Cochrane library and the Bibliothèque Nationale du Luxembourg (BnL). Due to different guideline characteristics for each data bank, various search syntaxes have been applied to form an adequate search strategy. While the search string in Pubmed goes with the following terms: (cardiac rehabilitation OR exercise training OR aerobic interval training) AND (coronary heart disease OR bypass surgery OR CABG OR coronary artery bypass grafting) AND (high-intensity interval training OR HIIT) AND (moderate-intensity continuous training OR MICE) AND (maximal oxygen consumption OR peak oxygen uptake OR 6MWT OR quality of life), the Cochrane library and the BnL use other keywords like (“rehabilitation” AND “coronary artery bypass surgery” AND “High-intensity interval training” AND “moderate-intensity continuous training”) respectively “cardiac rehabilitation” AND “high-intensity interval training” AND “moderate-intensity continuous training” AND “coronary artery bypass graft” AND “VO_2_peak” AND “quality of life” AND “6 min walking test”.

The manual database search was conducted until July 2022 and included all relevant published articles of the last 10 years.

### 2.5. Study Selection

Four group members (BS; LN; FL; EASR) independently conducted title and abstract screening considering potential inclusion and the topic. All the determined databases provided permanent access to all researchers, who then discussed and agreed on article selection before repeating independent screening on selected full texts regarding the previous identified inclusion/exclusion criteria. The final study inclusion was discussed and accepted by all team members. Finally, all included studies were analysed and assessed regarding their methodological quality.

All relevant information of the final article selection related to participants and their characteristics, interventions, methods, outcome measures, publication date, results and study design have been extracted, analysed and documented.

### 2.6. Quality Assessment

The methodological quality of the clinical trials was assessed using the Physiotherapy Evidence Database (PEDro) scale, reinforced by the use of the Cochrane Risk of Bias Tool for Randomised Trials for the evaluation of the risk of bias (RoB2), in order to provide more detail in the evaluation [19]. Latter one constitutes a literary recognised assessment method and allows subjective interpretation. The risk of bias evaluation was independently and manually conducted by the four reviewers (BS; LN; FL; EASR) using the RoB2 on the included articles of the present systematic review.

In addition, we calculated the kappa coefficient (κ) and the percentage of agreement scores to assess reliability before any consensus. Inter-rater reliability was estimated using κ > 0.7, indicating a high level of agreement between the reviewers, κ of 0.5–0.7, indicating a moderate level of agreement, and κ < 0.5 indicating a low level of agreement [20].

### 2.7. Certainty of Evidence

Different levels of evidence established the certainty of the evidence analysis according to the Grading of Recommendations, Assessment, Development and Evaluation (GRADE) framework, which has five domains: study design, imprecision, in-directness, inconsistency and publication bias [21]. The evidence classification followed four levels: high quality (all five domains are satisfied), moderate quality (one of the five domains is not satisfied), low quality (two of the five domains are not satisfied) or very low quality (three of five domains are not satisfied) [22].

For the risk of bias domain, recommendations were downgraded one level if there was an unclear or high risk of bias and severe limitations in the estimation effect. For consistency, recommendations were downgraded when point estimates varied widely among studies, confidence intervals overlapped or when the I2 test was substantial (>50%). For the indirectness domain, when significant differences in interventions, populations or outcomes were found, they were downgraded by one level. If there were fewer than 300 participants for key outcomes for the imprecision domain, it was downgraded to one level. Finally, if a strong influence of publication bias was detected, one level was downgraded [23].

## 3. Results

### 3.1. Results of the Search

**Figure 1:**
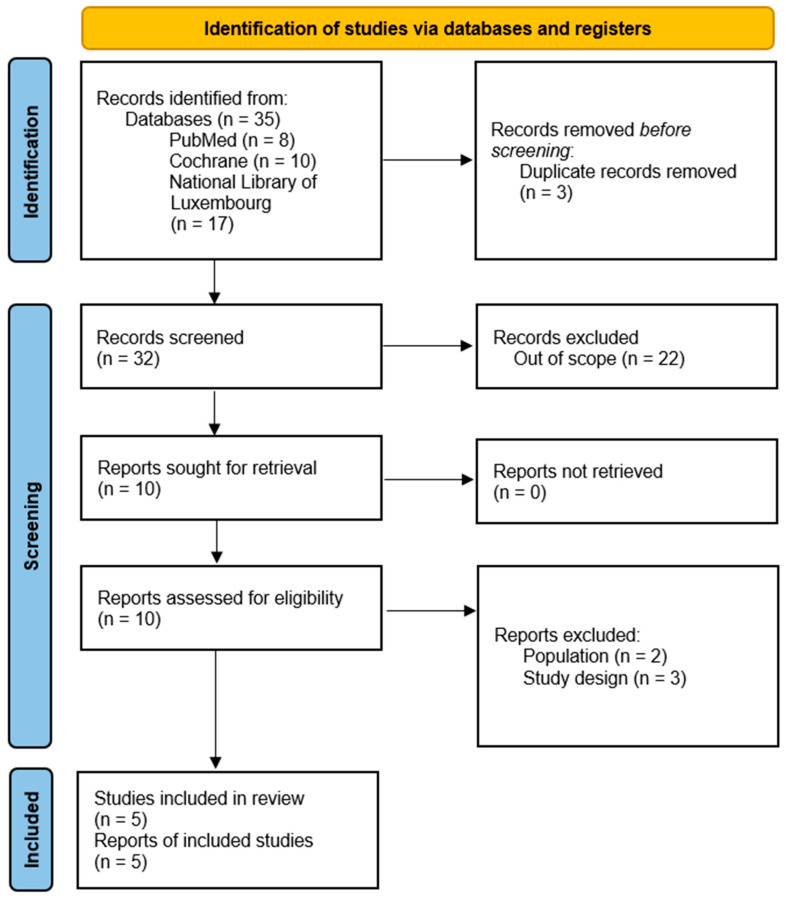
PRISMA 2020 flow diagram for new systematic reviews, including searches of databases and registers.

The initial electronic database search identified 35 articles, including eight from Pubmed, ten from the Cochrane Library and 17 from the BnL. Duplicates were removed, leading to 32 citations inserted for abstract screening. Post abstract screening, ten articles were retained for full-text eligibility, and only five were included in the systematic review, as represented in the PRISMA (Figure 1).

### 3.2. Excluded Studies

Five of 10 full-text screened articles have been excluded for two reasons. While 3 of them did not meet the outcome criteria (i.e., VO_2_ max, peakVO_2_, 6MWT and QoL assessments), two were rejected because of non-CABG participants (Figure 1).

### 3.3. Included Studies

Only five studies met the inclusion criteria, and all characteristics regarding participants, study type, year of production, training modalities, outcomes and results are summarised in Table 1.

### 3.4. Methodological Quality and Risk of Bias of the Included Studies

The methodological quality of the studies was evaluated with the PEDro scale, and the scores are shown in Table 2. One study was assessed as being of excellent quality [27], and four were of good quality [23,24,25,26,28].

**Figure 2:**
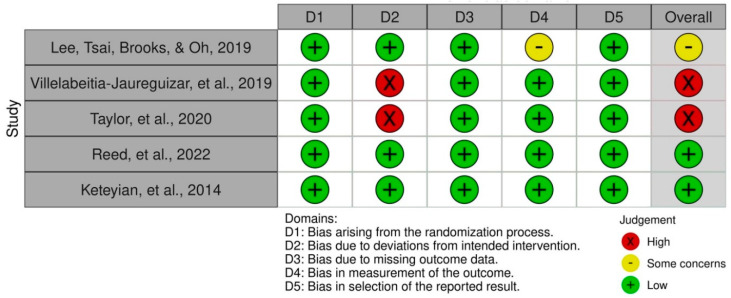
The evaluation regarding the risk of bias in the included studies [24,25,26,27,28].

Inter-examiner (BS; LN; FL; EASR) reliability had a high level of agreement (κ = 0.936) The risk of bias in the randomised controlled trials was evaluated with the Cochrane Risk of Bias Tool, and scores are shown in Table 3 and Figure 2. In total, five studies were evaluated. Two studies were at low risk of bias [26,27], one study had an unclear risk [23], and two studies were at high risk of bias [24,25]. Inter-examiner (BS; LN; FL; EASR) reliability had a high level of agreement (κ = 0.896).

Figure 2 demonstrates the evaluation regarding the risk of bias in the included studies and is presented in more detail in Supplementary Materials. Following results concerning the 5 main domains of the RoB2 can be reported for the 5 different RCTs:

- Randomisation & allocation process (selection bias): All 5 RCTs are judged to present a low risk of bias.
- Deviation from intended intervention (performance bias): Only 3 studies were classified with low risk related to this domain. The remaining two, namely the RCTs of Taylor et al. [28] and Villelabeitia-Jaureguizar et al. [25], present a high risk of bias due to different reasons. While one article reports a high non-adherence rate, which may affect the outcome results, the other one describes missing information about the awareness of the patients and their intervention and the lack of a possible adherence effect.
- Missing outcome (detection bias): All five studies were classified with a low risk of bias. However, Taylor et al. [28] reported some concerns as some outcome data were missing due to documentary reasons without influencing the outcomes.
- Measurement of the outcome (attrition bias): While 4 RCTs represent a low risk of bias, the study of Lee et al. [24] reveals some concerns due to missing information concerning the assessors.
- Selection of reported results (reporting bias): All 5 RCTs are considered low risk of bias.

### 3.5. Quality of Evidence

Quality of evidence of the effects of HIIT versus MICT on aerobic fitness and quality of life (QoL) in patients suffering from CABG was assessed with the Grading of Recommendations, Assessment, Development and Evaluation (GRADE) framework, and the results are shown in Table 4.

The moderate quality of the evidence supports the use of HIIT and MICT to improve aerobic fitness and quality of life (QoL). Inter-examiner (BS; LN; FL; EASR) reliability was a high level of agreement (κ = 0.829)

### 3.6. Effect of HIIT and MICT on Aerobic Fitness

Five studies analysed the effects of HIIT and MICT on aerobic fitness [24,25,26,27,28]. Most studies had excellent methodological quality, although the risk of bias ranged from high to low, with many studies scoring a high risk of bias [25,28] and only two presenting a low risk of bias [26,27].

Lee et al. [24] aimed to evaluate the effects of aerobic interval training (AIT) versus MICT on cardiorespiratory fitness with the peakVO_2_ as the primary outcome. All participants were exclusively women with CAD, including CABG and referred to a 24-week outpatient program (randomised to AIT or MICE). Baseline and post-24-week CR program assessments were performed using a metabolic card for the peakVO_2_, heart rate and speed data, respectively self-reported diaries for adherence. Due to the high drop-out rate resulting only in 14 participants completing the study, exists insufficient power to detect statistically significant differences between both group modalities. As stated by the authors, none of the reported differences regarding the various subsections is statistically significant unless explicitly indicated and introduced as hypotheses for future research. Stating the stats, post-program, only the primary outcome of peakVO_2_ was significantly higher in the AIT group compared to the MICT group (*p* = 0.036), showing a statistically significant improvement only for the AIT group after the intervention (*p* = 0.04). However, the significant treatment effect regarding the peakVO_2_ in the AIT group compared to the MICE (*p* < 0.001) was only demonstrated in the per-protocol analysis, while the intention-to-treat analysis provided no significant treatment effect of AIT on peakVO_2._ While the MICT participants completed 72.2% ± 15.2% of the exercise program, the AIT group absolved 76.2% ± 13.6% of their training sessions, showing no significant differences between groups (*p* > 0.05). However, measurements of the HR and RPE presented a mean peak HR intensity of 68% ± 7.3% and reported an RPE of 11.2 ± 1.3 on the Borg scale for the MICE. The AIT group exercised at a mean intensity of 88.5% ± 2.9 of peak heart rate and RPE of 16.7% ± 0.6 on the Borg scale being significantly higher (*p* < 0.01 for both).

In the year of 2019, Villelabeitia-Jaureguizar et al. [25] published an RCT intending to compare the effects of moderate continuous training (MCT) versus HIIT on mechanical efficiency (ME) in coronary patients. The 110 selected patients had different CAD, including CABG and were randomly assigned to either the MCT or HIIT to perform 24 training sessions over two months. Specific Cardiopulmonary exercise test (CPET) variables, namely the first and second ventilatory threshold (VT1 & VT2) and oxygen uptake (VO_2_), as well as the ME measurements, were collected at baseline and post-intervention. The effect evaluation of both training methods on the quantitative variables was performed by comparing the pre-and post-program values using the student-dependent samples *t*-test and expressed as mean and standard deviation. While the student *t*-test was applied to assess comparisons between the MICT and HIIT on quantitative variables, the x² test of association or Fisher exact test was used for qualitative variables. All comparisons were performed based on the two-tailed test, and the significance level was set at *p* < 0.05. The results of the cardiopulmonary exercise test, a significant increase in the areas of the peakVO_2_, exercise workload achieved and the total time of exercise effort can be reported in both groups with a more significant increase in the HIIT group (*p* < 0.05). VO_2_ at VT1 and VT2 demonstrated a significant augmentation in both groups with a higher increase in the HIIT participants (*p* < 0.05). While the power at VT1 significantly increased in both groups, with a more significant increase in the HIIT (*p* < 0.01), the power at VT2 only significantly increased in the HIIT training group (*p* < 0.001). Regarding the energy expenditure and ME values, the following results can be accentuated: VT1, VT2 and peakVO_2_ of energy expenditure demonstrate a significant increase in the post-exercise intervention compared to initial values for both training modalities with a significant increase in the HIIT group. Concerning measurements in ME, a significant increase in VT1 is reported for both groups, however with a higher increase for the HIIT group (*p* < 0.01), while the ME at peakVO_2_ and VT2 only significantly increased in the participants performing HIIT (*p* < 0.001).

In another study, Reed et al. [26] investigated and compared the effect of HIIT, Nordic Walking (NW) and MICT on functional capacity in patients diagnosed with CAD. All participants (N = 130) were randomly allocated to either the HIIT, NW or MICT group and performed supervised exercise sessions two times per week for 12 weeks. The baseline and 12-weeks follow-up assessment consisted of the 6MWT for functional capacity.

The results post-cardiac rehabilitation program showed a significant time effect that demonstrated an increased distance for the 6MWT (F = 138.736, *p* < 0.001). However, a significant time x group interaction was noticed, with a higher increase in the distance of the 6MWT for participants exercising in the NW group (F = 3.280, *p* = 0.042) compared to the MICT group (F = 4.962, *p*= 0.029) and HIIT (F = 4.039, *p* = 0.048). While 47% of patients in the HIIT group, 63% of the NW type and 38% of the MICT category achieved the minimally clinically significant difference, with no differences in these proportions being reported (x² = 4.299, *p* = 0.117).

Keteyan et al. [27] executed an RCT to compare the effects of HIIT and MCT on cardiorespiratory fitness using peakVO_2_ as the primary outcome. Thirty-nine participants with CAD, including CABG, were randomly assigned to one group and performed exercise training for ten more weeks in cardiac rehabilitation. Baseline and post-treatment assessments were conducted with cardiopulmonary exercise testing (CPX) using the modified-Bruce treadmill protocol. The comparison of group differences from baseline to follow-up post-treatment was reported using a student 2 sample *t*-test or a Welsch 2 sample *t*-test. *p* < 0.5 was accepted as significant. Stating the results, out of 21 HIIT members, 15 completed the program, while five patients dropped out in the MCT group. No differences were reported between groups after the follow-up comparing the heart rate and blood pressure at rest. However, the HIIT group presented a significantly more significant increase in submaximal endurance, measured by changes in VO_2_ at the ventilatory-derived anaerobic threshold (3.0 ± 2.8 mL· kg^−1^ · min^−1^, 21%) compared to MCT (0.7 ± 2.2 mL· kg^−1^ · min−1, 5%). Furthermore, both groups revealed lower submaximal heart rate at the CPX’s end of stage 2, with no significant differences between HIIT (−8 ± 7 beats· min^−1^) and MCT (−8 beats ± 7 · min^−1^). Lastly, after the follow-up, peak exercise capacity showed a higher improvement in the HIIT group (3.6 ± 3.1 mL· kg^−1^ · min^−1^, 16%) compared to MCT (1.7 ± 1.7 mL· kg^−1^ · min^−1^, 8%). Improvement in the total exercise duration and peak oxygen pulse was noted for both intervention groups, without significant differences. Overall, 67% of a participant performing HIIT showed an increase of peakVO_2_ of > 2 mL · kg^−1^ · min^−1^ in peakVO_2_, while this improvement was only noticed in 33% of MCT group members.

Taylor et al. [28] conducted a study investigating the effect of HIIT versus MICT on peakVO_2_ after a 4-week supervised and continuing 11 months home-based training program. Further intentions consisted of analysing the effect of both training modalities on feasibility, safety, adherence and cardiovascular risk factors. A total of 93 patients with CAD, including CABG, were randomly assigned to the HIIT or MICT group, while only 69 participants completed the entire study. Baseline, post-intervention and 3, 6 and 12 months follow-up assessment was performed using the CPET for peakVO_2_ and participant questionnaire regarding feasibility. Safety and anthropometric factors were continuously monitored during the study period, while adherence was evaluated as 70% attendance of the prescribed training sessions. After four weeks of a supervised rehabilitation program, a 10% increase of peakVO_2_ in the HIIT group and a 4% improvement for the MCT were revealed. Furthermore, for peakVO_2_ normalized for lean body mass, similar results can be reported: HIIT (4.1 [4.9] mL/kg/min, 10%) –MICT (1.0 [5.0] mL/kg/min, 2% improvement). Assessing post 12 months showed similar improvement regarding peakVo_2_ for both groups with HIIT (2.9 [4.5] mL/kg/min; 10%) and MICT 1.8 [4.3] mL/kg/min; 7%).

Nine serious incidents can be reported concerning the safety outcome, but none is due to the physician or the exercise program modalities. Feasibility was high for all participants, and no difference was presented for cardiovascular risk factors between groups apart from blood pressure, demonstrating a higher decrease in MICT patients compared to those in the HIIT group. Finally, adherence revealed the following statistics: average training RPE was greater for the HIIT than for the MICT group with (mean (SD) RPE: HIIT, 16.3 (1.3); MICT, 12.4 (0.6); *p* < 0.01). Average training heart rate as % of peak heart rate percentage was: 87% for HIIT and 71% for MICT and (*p* < 0.001). Adherence to the home-based program was maintained, but as the period continued, more adherence reduction was observed without differences between both groups.

### 3.7. Effect of of HIIT and MICT on QoL

Lee et al. [24], to evaluate the effects of aerobic interval training (AIT) versus MICT, also wanted to assess the effects of both exercise modalities concerning cognition, cardiovascular risk profile, adherence, and QoL.

These study variables were analysed based on self-reported diaries for adherence, Trail-making test part B & California verbal learning test for cognition and the medical health questionnaire regarding QoL. For the QoL, cognitive function and cardiovascular risk factors, no significant differences were revealed in the per-protocol analysis or the intention-to-treat analysis. Finally, adherence was high in both groups. While the MICT participants completed 72.2% ± 15.2% of the exercise program, the AIT group absolved 76.2% ± 13.6% of their training sessions, showing no significant differences between groups (*p* > 0.05).

Reed et al. [26] also investigated the different training modalities on depression severity, brain-derived neurotrophic factor (BDNF) and QoL. Baseline and 12-weeks follow-up assessment consisted of the Beck Depression Inventory-II (BDI-II) for depression, blood sample for BDNF, respectively the short-Form-36 (SF-36) version 1.0 and disease-specific QoL for QoL.

Regarding depression severity, other significant main effects of time (F = 14.700, *p* < 0.001) and group (F = 3.793, *p* = 0.025) reported improvements for BDI-II values plus lower overall depression severities scores in the group performing HIIT exercise compared to the MICT groups (*p* = 0.020). The Minimal Clinically Important Difference (MCID) of 5 points was achieved by 28% of HIIT participants, 28% of NW patients and 44% of MICT group members, showing no differences in these proportions between training modalities (x² = 2.118, *p* = 0.909). General and disease-specific QoL increased in HeartQol global (F = 12.908, *p* < 0.001), physical (F = 11.273, *p* = 0.001) and emotional (F = 21.239, *p* < 0.001) scores, demonstrating significant main effects of time with no main effects of groups being reported. Further main effects of time were reported for physical functioning (F = 17.448, *p* < 0.001), role limitations due to physical health (F = 20.822, *p* < 0.001), pain in body (F = 13.190, *p* < 0.001), general health (F = 27.192, *p* < 0.001), vitality (F = 25.913, *p* < 0.001), social functioning(F = 13.186, *p* < 0.001), role limitation because of emotional problems (F = 18.262, *p* < 0.001), mental health (F = 23.521, *p* < 0.001), mental component summary (MCS; F = 17.716, *p* < 0.001) and physical component summary (PCS; F = 18.664, *p* < 0.001) scores. 44%, 36% and 38% of the HIIT, NW, respectively MICT participants obtained the 5 points of the MCID for the MCS, revealing no differences in these proportions (x² =0.461, *p* = 0.749). The MCID score of 5 for PCS was attained by an adequate percentage number of HIIT, MICT and NW participants, also showing no differences in these proportions (x² = 1.222, *p* = 0.543). Finally, HIIT showed significantly higher vitality (*p* = 0.006), mental health (*p* = 0.023) and MCS (*p* = 0.025) scores compared to the MICT group.

Finally, in the study by Taylor et al. [28] investigating the effect of HIIT versus MICT, QoL improved in all training interventions without significant differences between both groups.

## 4. Discussion

The goal of the present systematic review was to compare and investigate the effects of HIIT and MICT on cardiorespiratory fitness and QoL in patients post CABG surgery. Although revascularisation, and more precisely CABG, is considered the most common surgical intervention worldwide [7], no international consensus for CR exists, leading to different mode, frequency and intensity training structures [9]. The present research demonstrates that both training methods provide beneficial outcomes regarding aerobic fitness and QoL with greater support and improvement in HIIT groups.

However, these results must be considered with caution, as this study contains several limitations that merit emphasis. Firstly, it is essential to accentuate the low availability and inclusion of studies in this literature research due to specific inclusion criteria. The main criterion was analysing the impact of two training modalities on cardiac patients undergoing CABG, representing a further limitation in this review. None of the 5 RCTs specifically investigated CABG patients but recruited participants suffering from different CADs, including CABG, whom all performed the same training instead of an established CABG protocol.

Furthermore, the high drop-out rate leads to smaller sample sizes than initially, and the gender distribution is another limitation in the result interpretation. Apart from the trial by Reed et al. [26], who exclusively recruited female participants to conduct their study, most eligible patients were men, which may limit the generalizability of the present findings. Although women’s leading cause of mortality is related to CAD, predominantly all studies compromise a low number of female participants, and little knowledge exists if the beneficial outcomes in men can also be applied to women [29].

The intervention period differs between all studies; no one investigated the training effect on long-term follow-up except for one. While the participants of Lee et al. [24] performed aerobic exercise for 24 weeks, Villelabeitia-Jaureguizar et al. [25] and Reed et al. [26] only required eight, respectively, 12 weeks of CR. Only Keteyan et al. [27], who added ten more supplementary weeks of CR to the initial program and Taylor et al. [28], who even demanded and executed a 12-month intervention and follow-up, investigated the effects on longer terms. Next to various periods of CR, the frequency differed from study to study, again underlining the missing protocol for structuring optimal CR. While Lee et al. [24] required a maximum of 5 sessions per week, the participants of Reed et al. [26] only performed two pieces of training per week, respectively 3 in Taylor et al. [28] and Villelabeitia-Jaureguizar et al. [25] trial. The content, the modality, the intensity and the length per session, however, presented some concordance and consistency among the studies. Nearly all sessions included a Warm-up, MICT or HIIT and a Cool-down, mostly performed on a treadmill, cycle ergometer or track. Furthermore, all sessions lasted between 40 and 60 min and were performed at an intensity of around 60% to 80% of peakVO_2_ or peak HR for MICT, respectively, at 90% to 95% peakVO_2_ or HR for HIIT participants. All HIIT interventions contained four intervals of high intensity for 4 min, followed by a 3-min active recovery.

Some researchers additionally demanded the participants to weekly execute home-based training to achieve the provided CR sessions, which is hard to control and may influence the outcomes.

Despite the numerous restrictions, most of the studies reported greater improvement in cardiorespiratory fitness and QoL in patients participating in the HIIT group, which contradicts the analysis of Trachsel et al. [30]. Latter, one conducted a study comparing HIIT and MICT on patients post revascularisation and evaluated MICT to be superior to HIIT concerning peakVO_2._ Also, Pearson et al. [31] mention their concern regarding HIIT, as the latter method is frequently perceived as more intense or stressful, generating insecurity in patients who are already predisposed to experience cardiac or adverse events. Furthermore, HIIT was frequently described as a training method which is less enjoyable and also more tiring, boring and demanding than MICT and is less applied due to lack of feasibility [32]. In the year 2020, Callum et al. [33] clarified the issue of insecurity by emphasising HIIT as a safe and effective training method, just for which, until today, no optimal protocol has been determined. Even though, until today, most CR guidelines prefer and recommend moderate-intensity exercise prescriptions to improve aerobic fitness [34], HIIT is nevertheless gaining popularity as a further training modality in CR programs [9]. However, as already stated by many authors, the latter and also the European Association of Preventive Cardiology (EAPC), together with the American Association of Cardiovascular and Pulmonary Rehabilitation and the Canadian Association of Cardiac Rehabilitation, demand further research and evidence on HIIT regarding feasibility, safety, adherence, long term follow up and different cardiac pathologies [34]. Due to the actuality that HIIT induces a more significant exercise stimulus with further increases in maximal aerobic capacity compared to MICT [35] and the growing interest in the scientific literature regarding HIIT in CR, the latter training method may have the chance to prevail against MICT if future research is conducted.

### 4.1. Future Directions

Although HIIT presents higher efficiency than MICT, further research with large scale-trials and specifically CABG patients need to be performed to provide the optimal training protocol for CR. The lack of information still exists and encourages further research for different HIIT domains like feasibility, safety, efficiency and long-term follow-ups. However, due to the enormous literary interest in HIIT, the latter training modality possesses high potential.

### 4.2. Limitations

Due to the small number of studies finally included in this systematic review, no results meta-analysis has been carried out, which should be considered a limitation. However, the systematic review carried out amply responds to the stated objectives.

Regardless of the exercise intervention, it should be noted that the included studies had a significant bias in allocation concealment during the development of the entire intervention, and participant/therapist blinding being the lowest scoring item. In contrast, the methodological quality of included studies was good to excellent in most of them.

Finally, the low quality of reporting in various studies, making it impossible to include them in the analysis, represents another limitation.

## 5. Conclusions

Both training methods improve cardiorespiratory fitness and QoL, with a more significant increase in HIIT. The moderate quality of evidence supports the use of HIIT and MICT to improve aerobic fitness and QoL. Although the positive outcomes support HIIT, further research is requested. High drop in our rates, low study availability and no trials in exclusively CABG patients generate more exploration regarding the topic.

## Figures and Tables

**Table 1:** The effects of HIIT versus MICT on aerobic fitness and quality of life (QoL) in patients suffering from CABG (description of included studies).

Author, Year.	Study Design	Aim of the Study	Participants	Treatment	Outcome Measures	Reported Results
Lee et al. (2019) [24]	Randomised controlled trial	To investigate the effects of AIT versus MICE on aerobic exercise capacity (VO_2_peak) in women CAD and who were referred to a large, 24-week outpatient CR programme. Secondary objectives included comparing the effects of AIT versus MICE on cognition, cardiovascular risk profile, adherence and quality of life before and after the 24-week CR programme.	31 patients > 50 years old, Female CAD patients (68.2 ± 9.2 years old) with: - Documented CAD -Left ventricular ejection fraction -3 weeks postmyocardial infarction (MI) or percutaneous coronary intervention -8 weeks postcoronary artery bypass graft	-MICE: track or treadmill for 30–40 min with the intensity of 60%–80% of peakVO_2_ + warm up and cool down period -AIT: 6 weeks run in (MICE) followed by 3 AIT sessions and 2 MICE sessions per week. (AIT: warm-up 10–15 min of 60%–70% peak heart rate, then four 4-min intervals at 90%–95% of peak HR with 3 min active recovery at 50%–70% peak heart rate and a cool-down period	-peakVO_2_ -Cardiovascular risk factors - Adherence -Quality of life -Cognitive assessment	-Unanticipated challenges in recruitment availability and eligibility, in combination with a 59% and 50% attrition rate in the AIT and MICE groups, respectively, rendered the study underpowered to detect differences between groups. -The treatment effect analysis, however, unveiled a 0.95 mL kg^−1^ min^−1^ improvement in peakVO_2_ in response to AIT over MICE (*p* < 0.001)
Villelabeitia-Jaureguizar et al. (2019) [25]	Randomised controlled trial	To compare the influence of two different exercise protocols: MCT versus HIIT, as part of a CR program on ME values among coronary patients	110 patients were studied (53 patients in MCT-group and 57 patients in HIIT-group; 58.3 ± 9.5 vs 57.6 ± 9.8 years old) with stable New York Heart Association functional class I or II CAD with angina pectoris or MI, or cardiac event, elective percutaneous coronary intervention, CABG post 6–12 weeks	-Randomized 1 to 1 in either HIIT or MICE cycle ergometer training 3 times per week over 2 months (24 sessions) -First weeks HR reached at VT1, afterwards VT1 + 10% -The steep ramp test (SRT) protocol used to determine HIIT-program →first month 20 s repetitions at 50% of the maximum workload reached with the SRT, then 40 s recovery at 10% →2nd month: analysing results of new SRT	Energy consumption and mechanical efficiency calculations were obtained at intensities corresponding to VT1, VT2 and at peakVO_2_	-Higher peakVO_2_ increase in the HIIT group (2.96 ± 2.33 mL/kg/min vs 3.88 ± 2.40 mL/kg/min, for patients of the MCT and HIIT groups, respectively, *p* < 0.001). -Greater ME increase at VT1 in the HIIT group (2.20 ± 6.25% vs 5.52 ± 5.53%, for patients of the MCT and HIIT groups, respectively, *p* < 0.001). - The application of HIIT to patients with chronic ischemic heart disease of low risk resulted in a greater improvement in peakVO_2_ and ME at VT1 than when MCT was applied
Reed et al. (2022) [26]	Randomised controlled trial	To compare the effects of 12 weeks of HIIT, NW and MICT on functional capacity in CAD patients. The effects on depression severity, BDNF and QoL were also examined	135 patients with CAD (aged 61 ± 7 years; male: 85%) who underwent CABG or PCI within the last 18 weeks or patients referred to the CR program	Randomised 1:1:1 either to HIIT, MICT or NW and performed 2 times per week over 12 weeks -HIIT (cycle ergometer, treadmill, elliptical, dance: 45 min each session with 10 min warm-up at 60–70% peak HR, 4 × 4 min HIIT at 85%–95% peak HR interspersed with 3 min of low-intensity periods with 60%–70% peak HR and a cool-down period of 60%–70% peak HR with stretching and strength exercises -NW: 60 min each session with 15 min warm-up, 10–15 min of continuous/intermittent walking with NW poles for the first three weeks, with progress to 30 min for the remaining weeks and a cool-down period of 15 min with stretching -MICT: 60 min with 10–15 min of warm-up walking or aerobic equipment, 10–15 min of continuous aerobic conditioning in the first 3 weeks (walking, jogging, cyclo ergometer, rowing), then progressing to 30 min for the last weeks and 15 min of a cool-down period with stretching and strength exercises	-Functional capacity (6MWT) -Depressive symptoms -Quality of life -Anthropometrics and hemodynamics -Brain-derived neurotrophic factor	-Better results in NW than HIIT and MCE -Greater increases in 6MWT distance (m) for NW (77.2 ± 60.9) than HIIT (51.4 ± 47.8) and MICT (48.3 ± 47.3) -BDI-II significantly improved (HIIT: −1.4 ± 3.7, NW: −1.6 ± 4.0, MICT: −2.3 ± 6.0 points, main effect of time: *p* < 0.001 -SF-36 and HeartQoL values were observed (main effects of time: *p* < 0.05). HIIT, NW and MICT participants attended 17.7 ± 7.5, 18.3 ± 8.0 and 16.1 ± 7.3 of the 24 exercise sessions, respectively (*p* = 0.387).
Ketevian et al. (2014) [27]	Randomised controlled trial	The study tested the hypothesis that HIIT could be deployed into a standard CR setting and would result in a greater increase in cardiorespiratory fitness (i.e., peak oxygen uptake, VO_2_) versus MCT.	−39 patients (23 men; mean age 68 ± 8 years) with sinus rhythm; ejection fraction > 40%; >3 weeks following myocardial infarction or percutaneous coronary intervention, >4 weeks after CABG -Were randomised to HIIT (mean age 60 ± 7 years) and 13 to MICT (mean age 58 ± 9 years)	-Randomized 1:1 to HIIT or MCT for additional 10 more weeks of CR of their initial CR -MCT: 5 min active warm-up, 30 min cardiorespiratory training on treadmill with 60%-80% HR reserve, 5 min active cool down -HIIT (treadmill): 5 min warm-up, followed by 3 min of 60%–70% of HR reserve training, followed by 4 HIIT work intervals of 4 min each with 80%–90% HR reserve. Then active recovery period of 60%–70% of HR reserve followed again by the 4 higher intervals with finally a cool-down period	-Blood pressure -Heart rate at stage 2 of exercise test -Oxygen uptake at ventilatory derived anaerobic threshold -Peak oxygen pulse (mL · beat^−1^) -Peak respiratory exchange ratio - VE-CO_2_ -Change in heart rate from peak exercise to minute 1 of recovery, bpm, peakVO_2_	-VO_2_ at ventilatory-derived anaerobic threshold increased more with HIIT: 3.0 ± 2.8 mL · kg^−1^ · min^−1^, 21%) compared to MCT (0.7 ± 2.2 mL· kg^−1^ · min^−1^, 5%); *p* < 0.05. -Peak VO_2_ improved better in HIIT group HIIT versus MCT (3.6 ± 3.1 mL· kg^−1^·min^−1^ vs 1.7 ± 1.7 mL· kg^−1^·min^−1^; *p* < 0.05). -HIIT resulted in a more significant improvement in maximal exercise capacity and submaximal endurance than MCT.
Taylor et al. (2020) [28]	Randomised controlled trial	To compare HIIT with MICT for feasibility, safety, adherence, and efficacy of improving VO_2_ peak in patients with CAD	96 patients (78 men; mean age 68 ± 8 years); 46 were randomised to HIIT and 47 to MICT during 4-week supervised program) with angiographically proven CAD	-1:1 randomisation to HIIT or MICT and performed 3 sessions per week (1 supervised; 2 home-based) for 4 weeks and then continue for 11 further months 3 times per week -HIIT: 4x4 -minute high-intensity intervals of 15–18 (RPE) or 15–18 on the Borg scale 6–20 interspersed with 3-min active recovery intervals -MICT: 40 min at an RPE of 11–13.	- peakVO_2_ --Feasibility -Safety -Adherence -Cardiovascular risk factors -Quality of life	-After 4 weeks, HIIT improved peakVO2 by 10% compared with 4% in the MICT group -HIIT, 2.9 [3.4] mL/kg/min; MICT, 1.2 [3.4] mL/kg/min; *p* = 0.02) -After 12 months, there were similar improvements from baseline between groups, with a 10% improvement in the HIIT group and a 7% improvement in the MICT group (HIIT, 2.9 [4.5] mL/kg/min; MICT, 1.8 [4.3] mL/kg/min; *p* = 0.30)

Abbreviations alphabetically ordered: Aerobic Interval Training (AIT); Banque Nationale de Luxembourg (BnL); Brain-Derived Neurotrophic Factor (BDNF); British Association for Cardiovascular Prevention and Rehabilitation (BACPR); Beck Depression Inventory-II (BDI-II); Cardiovascular Disease (CVD); Cardiac Rehabilitation (CR); Coronary Artery Bypass Graft Surgery (CABG); Coronary Artery Diseases (CAD); Cardiopulmonary Exercise Test (CPET); Heart Rate (HR); High Intensity Interval Training (HIIT); Moderate Intensity Continuous Training (MICT; MCT); Mechanical Efficiency (ME); Moderate Intensity Cardio Exercise (MICE); 6 Minutes Walking Test (6MWT); National Institute for Health and Care Excellence (NICE); Nordic Walking (NW); Quality of Life (QoL); Rate of Perceived Exertion (RPE); Revised Cochrane Risk-of-Bias Tool for Randomised Trials (RoB2); Short-Form-36 (SF-36); First and Second Ventilatory Threshold (VT1 & VT2); Oxygen Uptake (*VO_2_*); Peak Oxygen Uptake (peakVO_2_); Maximal Oxygen Consumption (VO_2_max); Ventilatory Efficiency Slope (VE-CO_2_); World Health Organization (WHO).

**Table 2:** Methodological quality evaluation of the clinical trials using the PEDro scale for randomized trials.

Scale (Physiotherapy Evidence Database (PEDro)) to Analyze the Methodological Quality of Clinical Studies												
Authors	Specified Selection Criteria	Randomization	Hidden Assignment	Similar Groups to Start	Blinded Subjects	Blinded Therapists	Blinded Raters	Outcomes 85%	Treatment or Intention to Treat	Comparison between Groups	Point Measures Variability	Outcome
Lee et al. (2019) [24]	Yes	Yes	Yes	Yes	No	No	No	No	Yes	No	Yes	6
Villelabeitia-Jaureguizar et al. (2019) [25]	Yes	Yes	Yes	Yes	No	No	Yes	Yes	Yes	No	Yes	8
Reed et al. (2022) [26]	Yes	Yes	Yes	Yes	No	No	No	Yes	Yes	Yes	Yes	8
Ketevian et al. (2014) [27]	Yes	Yes	Yes	Yes	No	Yes	Yes	No	Yes	Yes	Yes	9
Taylor et al. (2020) [28]	Yes	Yes	No	Yes	No	No	No	Yes	Yes	Yes	Yes	7

Result on the PEDro scale: 9–10 (excellent), 6–8 (good), 4–5 (acceptable) and <4 (poor).

**Table 3:** Methodological quality evaluation of the clinical trials using the Cochrane Risk of Bias Tool for assessing the risk of bias in randomized trials.

Cochrane Risk of Bias Collaboration Tool for Randomized Controlled Trials							
Author (Year)	Random Sequence Generation	Allocation Concealment	Blinding (Participants and Personnel)	Blinding (Outcome Assessment)	Incomplete Outcome Data	Selective Reporting	Other Sources of Bias
Lee et al. (2019) [24]	Low risk	Low risk	High risk	High risk	High risk	Low risk	Low risk
Villelabeitia-Jaureguizar et al. (2019) [25]	Low risk	Low risk	High risk	Low risk	Low risk	Low risk	Low risk
Reed et al. (2022) [26]	Low risk	Low risk	High risk	High risk	Low risk	Low risk	Low risk
Ketevian et al. (2014) [27]	Low risk	Low risk	Unclear	Low risk	Low risk	Low risk	Low risk
Taylor et al. (2020) [28]	Low risk	High risk	High risk	High risk	Low risk	Low risk	Low risk

**Table 4:** Summary of findings for clinical trials using the GRADE quality of evidence assessment.

**Quality Assessment of Studies on HIIT Improving Aerobic Fitness and QoL**							
**Number of Studies (Subjects)**	**Risk of Bias**	**Inconsistency**	**Indirectness**	**Imprecision**	**Publication Bias**	**Quality**	**Grade of Recommendation**
5 (*n* = 168)	Serious *	Not serious †	Not serious	Not serious	Not serious †	Moderate quality	Strongly in favor
**Quality Assessment of Studies on MICT Improving Aerobic Fitness and QoL**							
**Number of Studies (Subjects)**	**Risk of Bias**	**Inconsistency**	**Indirectness**	**Imprecision**	**Publication Bias**	**Quality**	**Grade of Recommendation**
5 (*n* = 169)	Serious *	Not serious †	Not serious	Not serious	Not serious †	Moderate quality	Strongly in favor

* Blinding and/or allocation concealment issues. † Low number of participants. The GRADE system establishes four degrees of evidence (high, moderate, low and very low) and two degrees of recommendation (strong or weak) for or against an intervention. For each item, a judgment is made (very serious, serious, not serious).

## Data Availability

The data presented in this study are available on request from the corresponding authors.

## Supplementary Materials

The following are available online at https://www.mdpi.com/article/10.3390/jcdd9100328/s1. References [24,25,26,27,28] are cited in the supplementary materials.
