# Supplementary material for: The Comparison of High-Intensity Interval Training Versus Moderate-Intensity Continuous Training after Coronary Artery Bypass Graft: A Systematic Review of Recent Studies
Source: J Cardiovasc Dev Dis. 2022 Sep 28;9(10):328. doi: 10.3390/jcdd9100328 (PMC9604034; doi:10.3390/jcdd9100328)
Supplement: Supplementary file 1 [file jcdd-09-00328-s001.zip › jcdd-1876356-supplementary.pdf]

Supplementary Material

Revised Cochrane risk-of-bias tool for randomized trials (RoB 2)

23. Lee, L. S., Tsai, M. C., Brooks, D., et al., (2019). Randomised controlled trial in women with coronary artery disease investigating the effects of aerobic interval training versus moderate intensity continuous exercise in cardiac rehabilitation: CAT versus MICE study. BMJ Open Sport &; Exercise Medicine, 5(1), e000589. https://doi.org/10.1136/bmjsem-2019-000589

Domain 1: Risk of bias arising from the randomization process

Signaling questions	Comments	Response
Was the allocation sequence random?	Patients were randomized 1:1, using an eight-block randomization allocation that a third party, independent researcher with no affiliations to the study generated using a computerized random number generator.	**YES**
Was the allocation sequence concealed until participants were enrolled and assigned to interventions?	Authors were blinded to the details of randomization allocation until study completion and were blinded to group allocation until after the collection of baseline data. Study participants were also blinded to group allocation until after the collection of baseline data was completed.	**YES**
Did baseline differences between intervention groups suggest a problem with the randomization process?	Despite the prospective, randomized approach employed in this study, the small sample size and random chance alone account for the overall differences between the groups.	**Probably NO**
Risk-of-bias judgement		**LOW RISK**
Optional: What is the predicted direction of bias arising from the randomization process?		

Domain 2: Risk of bias due to deviations from the intended interventions (effect of assignment to intervention)

Signaling questions	Comments	Response
2.1 Were participants aware of their assigned intervention during the trial?		**YES**
2.2 Were carers and people delivering the interventions aware of participant assigned intervention during the trial?		**YES**
2.3. If Y/PY/NI to 2.1 or 2.2: Were there deviations from the intended		**Probably NO**

intervention that arose because of the trial context?		
2.4 If Y/PY to 2.3: Were these deviations likely to have affected the outcome?		**NA**
2.5. If Y/PY/NI to 2.4: Were these deviations from intended intervention balanced between groups?		**NA**
2.6 Was an appropriate analysis used to estimate the effect of assignment to intervention?	The main analyses were performed using the intention-to-treat (ITT) principle, in which all patients were analyzed according to their initially assigned group of AIT or MICE at baseline, irrespective of study adherence or completion.	**YES**
2.7 If N/PN/NI to 2.6: Was there potential for a substantial impact (on the result) of the failure to analyze participants in the group to which they were randomized?		**NA**
Risk-of-bias judgement		**LOW RISK**
Optional: What is the predicted direction of bias due to deviations from intended interventions?		

Domain 2: Risk of bias due to deviations from the intended interventions (effect of adhering to intervention)

Signaling questions	Comments	Response
2.1. Were participants aware of their assigned intervention during the trial?		**YES**
2.2. Were carers and people delivering the interventions aware of participants assigned intervention during the trial?		**YES**
2.3. [If applicable:] If Y/PY/NI to 2.1 or 2.2: Were important non-protocol interventions balanced across intervention groups?		**NA**
2.4. [If applicable:] Were there failures in implementing the intervention that could have affected the outcome?		**NO**
2.5. [If applicable:] Was there non-adherence to the assigned intervention regimen that could have affected participants' outcomes?	Adherence was high. On average, patients completed 72.2%±15.2% of the five exercise sessions prescribed per week for the 24-week period in the MICE group, and the AIT group completed 76.2%±13.6% of their 5 weekly exercise sessions, with no significant differences between groups.	**NO**
2.6. If N/PN/NI to 2.3, or Y/PY/NI to 2.4 or 2.5: Was an appropriate analysis		**NA**

used to estimate the effect of adhering to the intervention?		
Risk-of-bias judgement		**LOW RISK**
Optional: What is the predicted direction of bias due to deviations from intended interventions?		

Domain 3: Missing outcome data

Signaling questions	Comments	Response
3.1 Were data for this outcome available for all, or nearly all, participants randomized?		**YES**
3.2 If N/PN/NI to 3.1: Is there evidence that the result was not biased by missing outcome data?		**NA**
3.3 If N/PN to 3.2: Could missingness in the outcome depend on its true value?		**NA**
3.4 If Y/PY/NI to 3.3: Is it likely that missingness in the outcome depended on its true value?		**NA**
Risk-of-bias judgement		**LOW RISK**
Optional: What is the predicted direction of bias due to missing outcome data?		

Domain 4: Risk of bias in measurement of the outcome

Signaling questions	Comments	Response
4.1 Was the method of measuring the outcome inappropriate?		**NO**
4.2 Could measurement or ascertainment of the outcome have differed between intervention groups?		**NO**
4.3 If N/PN/NI to 4.1 and 4.2: Were outcome assessors aware of the intervention received by study participants?		**NI**

4.4 If Y/PY/NI to 4.3: Could assessment of the outcome have been influenced by knowledge of intervention received?		**Probably NO**
4.5 If Y/PY/NI to 4.4: Is it likely that assessment of the outcome was influenced by knowledge of intervention received?		**NA**
Risk-of-bias judgement		**Some concerns**
Optional: What is the predicted direction of bias in measurement of the outcome?		

Domain 5: Risk of bias in selection of the reported result

Signaling questions	Comments	Response
5.1 Were the data that produced this result analyzed in accordance with a pre-specified analysis plan that was finalized before unblinded outcome data were available for analysis?		**YES**
Is the numerical result being assessed likely to have been selected, on the basis of the results, from...		
5.2. ... multiple eligible outcome measurements (eg. scales, definitions, time points) within the outcome domain?		**Probably NO**
5.3 ... multiple eligible analyses of the data?		**Probably NO**
Risk-of-bias judgement		**LOW RISK**
Optional: What is the predicted direction of bias due to selection of the reported result?		

Overall risk of bias: Some concerns

24. Villelabeitia-Jaureguizar, K., Vicente-Campos, D., Berenguel Senen, A., et al., (2013). Mechanical efficiency of high versus moderate intensity aerobic exercise in coronary heart disease patients: A randomized clinical trial. Cardiology Journal. https://doi.org/10.5603/cj.a2018.0052

Domain 1: Risk of bias arising from the randomization process

Signaling questions	Comments	Response
**1.1 Was the allocation sequence random?**	Patients were randomized on a one-to-one basis to either the MCT or the HIIT group.	**YES**
**1.2 Was the allocation sequence concealed until participants were enrolled and assigned to interventions?**	CPET were administered by staff who were unaware of the exercise training group the patients were assigned.	**YES**
**1.3 Did baseline differences between intervention groups suggest a problem with the randomization process?**		**No Information**
**Risk-of-bias judgement**		**LOW RISK**
Optional: What is the predicted direction of bias arising from the randomization process?		

Domain 2: Risk of bias due to deviations from the intended interventions (effect of assignment to intervention)

Signaling questions	Comments	Response
2.1. Were participants aware of their assigned intervention during the trial?		**No Information**
2.2. Were carers and people delivering the interventions aware of participants' assigned intervention during the trial?		**NO**
2.3. If Y/PY/NI to 2.1 or 2.2: Were there deviations from the intended intervention that arose because of the trial context?		**NO**
2.4 If Y/PY to 2.3: Were these deviations likely to have affected the outcome?		**NA**
2.5. If Y/PY/NI to 2.4: Were these deviations from intended intervention balanced between groups?		**NA**

2.6 Was an appropriate analysis used to estimate the effect of assignment to intervention?		**No Information**
2.7 If N/PN/NI to 2.6: Was there potential for a substantial impact (on the result) of the failure to analyse participants in the group to which they were randomized?		**Probably NO**
Risk-of-bias judgement		**Some Concerns**
Optional: What is the predicted direction of bias due to deviations from intended interventions?		

Domain 2: Risk of bias due to deviations from the intended interventions (effect of adhering to intervention)

Signaling questions	Comments	Response
2.1. Were participants aware of their assigned intervention during the trial?		**No Information**
2.2. Were carers and people delivering the interventions aware of participants' assigned intervention during the trial?		**NO**
2.3. [If applicable:] If Y/PY/NI to 2.1 or 2.2: Were important non-protocol interventions balanced across intervention groups?		**No Information**
2.4. [If applicable:] Were there failures in implementing the intervention that could have affected the outcome?		**NO**
2.5. [If applicable:] Was there non-adherence to the assigned intervention regimen that could have affected participants' outcomes?		**NO**
2.6. If N/PN/NI to 2.3, or Y/PY/NI to 2.4 or 2.5: Was an appropriate analysis used to estimate the effect of adhering to the intervention?		**No Information**
Risk-of-bias judgement		**High Risk**
Optional: What is the predicted direction of bias due to deviations from intended interventions?		

Domain 3: Risk of bias due to missing outcome data

Signaling questions	Comments	Response
3.1 Were data for this outcome available for all, or nearly all, participants randomized?		**YES**
3.2 If N/PN/NI to 3.1: Is there evidence that the result was not biased by missing outcome data?		**NA**
3.3 If N/PN to 3.2: Could missingness in the outcome depend on its true value?		**NA**
3.4 If Y/PY/NI to 3.3: Is it likely that missingness in the outcome depended on its true value?		**NA**
Risk-of-bias judgement		**LOW RISK**
Optional: What is the predicted direction of bias due to missing outcome data?		

Domain 4: Risk of bias in measurement of the outcome

Signaling questions	Comments	Response
4.1 Was the method of measuring the outcome inappropriate?	VO2 was determined breath by breath using an automated system (UltimaCardiO2, Medical Graphics Corporation, St. Paul, Minnesota, USA).<br>VT1 and VT2 were determined following the method of ventilatory equivalents described by Skinner et al.	**NO**
4.2 Could measurement or ascertainment of the outcome have differed between intervention groups?		**Probably NO**
4.3 If N/PN/NI to 4.1 and 4.2: Were outcome assessors aware of the intervention received by study participants?	CPET were administered by staff who were unaware of the exercise training group the patients were assigned.	**NO**
4.4 If Y/PY/NI to 4.3: Could assessment of the outcome have been influenced by knowledge of intervention received?		**NA**
4.5 If Y/PY/NI to 4.4: Is it likely that assessment of the outcome was influenced by knowledge of intervention received?		**NA**

Risk-of-bias judgement		**LOW RISK**
Optional: What is the predicted direction of bias in measurement of the outcome?		

Domain 5: Risk of bias in selection of the reported result

Signaling questions	Comments	Response
5.1 Were the data that produced this result analysed in accordance with a pre-specified analysis plan that was finalized before unblinded outcome data were available for analysis?	Plan listed in statistical analysis.	**YES**
Is the numerical result being assessed likely to have been selected, on the basis of the results, from...		
5.2. ... multiple eligible outcome measurements (eg. scales, definitions, time points) within the outcome domain?	All eligible reported results for the outcome domain correspond to all intended outcome measurements.	**Probably NO**
5.3 ... multiple eligible analyses of the data?		**Probably NO**
Risk-of-bias judgement		**LOW RISK**
Optional: What is the predicted direction of bias due to selection of the reported result?		

Overall risk of bias: High risk

27. Taylor, J. L., Holland, D. J., Keating, S. E., et al., (2020). Short-term and Long-term Feasibility, Safety, and Efficacy of High-Intensity Interval Training in Cardiac Rehabilitation. JAMA Cardiology, 5(12), 1382. https://doi.org/10.1001/jamacardio.2020.3511

Domain 1: Risk of bias arising from the randomization process

Signaling questions	Comments	Response
1.1 Was the allocation sequence random?	Participants underwent 1:1 randomization to either HIIT or MICT groups.	**YES**
1.2 Was the allocation sequence concealed until participants were enrolled and assigned to interventions?	The randomized sequence was computer-generated and sealed in sequentially numbered opaque envelopes by an individual external from the investigation team. A study investigator then enrolled participants and assigned them to interventions as per the numbered envelopes.	**YES**
1.3 Did baseline differences between intervention groups suggest a problem with the randomization process?	No imbalances apparent in baseline differences.	**YES**
Risk-of-bias judgement		**LOW RISK**
Optional: What is the predicted direction of bias arising from the randomization process?		

Domain 2: Risk of bias due to deviations from the intended interventions (effect of assignment to intervention)

Signaling questions	Comments	Response
2.1. Were participants aware of their assigned intervention during the trial?	C.F 1.2	**NO**
2.2. Were carers and people delivering the interventions aware of participants' assigned intervention during the trial?	C.F 1.2	**NO**
2.3. If Y/PY/NI to 2.1 or 2.2: Were there deviations from the intended intervention that arose because of the trial context?		**NA**
2.4 If Y/PY to 2.3: Were these deviations likely to have affected the outcome?		**NA**
2.5. If Y/PY/NI to 2.4: Were these deviations from intended intervention balanced between groups?		**NA**

2.6 Was an appropriate analysis used to estimate the effect of assignment to intervention?	For all outcomes, intention-to-treat analyses using linear mixed modelling were performed to investigate the interaction of time and group effects.<br>Prespecified per-protocol analyses were conducted including only participants meeting the criteria for exercise adherence.	**Probably NO**
2.7 If N/PN/NI to 2.6: Was there potential for a substantial impact (on the result) of the failure to analyze participants in the group to which they were randomized?		**NI**
Risk-of-bias judgement		**HIGH RISK**
Optional: What is the predicted direction of bias due to deviations from intended interventions?		

Domain 2: Risk of bias due to deviations from the intended interventions (effect of adhering to intervention)

Signaling questions	Comments	Response
2.1. Were participants aware of their assigned intervention during the trial?	C.F 1.2	**NO**
2.2. Were carers and people delivering the interventions aware of participants' assigned intervention during the trial?	C.F 1.2	**NO**
2.3. [If applicable:] If Y/PY/NI to 2.1 or 2.2: Were important non-protocol interventions balanced across intervention groups?		**NA**
2.4. [If applicable:] Were there failures in implementing the intervention that could have affected the outcome?	Implementation of the intervention was successful for most participants.	**NO**
2.5. [If applicable:] Was there non-adherence to the assigned intervention regimen that could have affected participants' outcomes?	The proportion who did not adhere is high enough to raise concerns.	**Probably YES**
2.6. If N/PN/NI to 2.3, or Y/PY/NI to 2.4 or 2.5: Was an appropriate analysis used to estimate the effect of adhering to the intervention?	Prespecified per-protocol analyses were conducted including only participants meeting the criteria for exercise adherence.	**Probably NO**
Risk-of-bias judgement		**HIGH RISK**
Optional: What is the predicted direction of bias due to deviations from intended interventions?		

Domain 3: Risk of bias due to missing outcome data

Signaling questions	Comments	Response
3.1 Were data for this outcome available for all, or nearly all, participants randomized?		**NO**
3.2 If N/PN/NI to 3.1: Is there evidence that the result was not biased by missing outcome data?		**NO**
3.3 If N/PN to 3.2: Could missingness in the outcome depend on its true value?	Missing outcome data occurred for documented reasons that are unrelated to the outcome (non-adherence).	**NO**
3.4 If Y/PY/NI to 3.3: Is it likely that missingness in the outcome depended on its true value?		**NA**
Risk-of-bias judgement		**LOW RISK**
Optional: What is the predicted direction of bias due to missing outcome data?		

Domain 4: Risk of bias in measurement of the outcome

Signaling questions	Comments	Response
4.1 Was the method of measuring the outcome inappropriate?	The primary outcome (VO2 peak) was measured by CPET.	**NO**
4.2 Could measurement or ascertainment of the outcome have differed between intervention groups?		**Probably NO**
4.3 If N/PN/NI to 4.1 and 4.2: Were outcome assessors aware of the intervention received by study participants?		**NI**
4.4 If Y/PY/NI to 4.3: Could assessment of the outcome have been influenced by knowledge of intervention received?		Probably NO
4.5 If Y/PY/NI to 4.4: Is it likely that assessment of the outcome was influenced by knowledge of intervention received?		**NA**

Risk-of-bias judgement		**LOW RISK**
Optional: What is the predicted direction of bias in measurement of the outcome?		

Domain 5: Risk of bias in selection of the reported result

Signaling questions	Comments	Response
5.1 Were the data that produced this result analyzed in accordance with a pre-specified analysis plan that was finalized before unblinded outcome data were available for analysis?	Pre-specified intentions planned outcome measurements and statistical analyses are available in sufficient detail.	YES
Is the numerical result being assessed likely to have been selected, on the basis of the results, from...		
5.2. ... multiple eligible outcome measurements (e.g. scales, definitions, time points) within the outcome domain?		**NO**
5.3 ... multiple eligible analyses of the data?	All eligible reported results for the outcome measurement correspond to all intended analyses.	**NO**
Risk-of-bias judgement		**LOW RISK**
Optional: What is the predicted direction of bias due to selection of the reported result?		

Overall risk of bias: High risk

25. Reed, J. L., Terada, T., Cotie, L. M., et al., (2022). The effects of high-intensity interval training, Nordic walking and moderate-to-vigorous intensity continuous training on functional capacity, depression and quality of life in patients with coronary artery disease enrolled in cardiac rehabilitation: A randomized controlled trial (CRX study). Progress in Cardiovascular Diseases, 70, 73-83. https://doi.org/10.1016/j.pcad.2021.07.002

Domain 1: Risk of bias arising from the randomization process

Signaling questions	Comments	Response
1.1 Was the allocation sequence random?	Patients were randomized in a 1:1:1 ratio using a blocked, stratified, random sequence that was computer-generated.	**YES**
1.2 Was the allocation sequence concealed until participants were enrolled and assigned to interventions?	Treatment assignments were placed in sealed, numbered opaque envelopes to ensure concealment until baseline data were collected.	**YES**
1.3 Did baseline differences between intervention groups suggest a problem with the randomization process?	Baseline characteristics were compared between groups to identify any chance differences that may have occurred despite random assignment	**NO**
Risk-of-bias judgement		**LOW RISK**
Optional: What is the predicted direction of bias arising from the randomization process?		

Domain 2: Risk of bias due to deviations from the intended interventions (effect of assignment to intervention)

Signaling questions	Comments	Response
2.1. Were participants aware of their assigned intervention during the trial?	The HIIT, NW and MICT sessions were performed at separate times to avoid contamination between groups.	**NO**
2.2. Were carers and people delivering the interventions aware of participants' assigned intervention during the trial?		**NO**
2.3. If Y/PY/NI to 2.1 or 2.2: Were there deviations from the intended intervention that arose because of the trial context?		**NA**
2.4 If Y/PY to 2.3: Were these deviations likely to have affected the outcome?		**NA**
2.5. If Y/PY/NI to 2.4: Were these deviations from intended intervention balanced between groups?		**NA**
2.6 Was an appropriate analysis used to estimate the effect of assignment to intervention?	An intention-to-treat analysis was used.	**YES**

2.7 If N/PN/NI to 2.6: Was there potential for a substantial impact (on the result) of the failure to analyze participants in the group to which they were randomized?		**NA**
Risk-of-bias judgement		**LOW RISK**
Optional: What is the predicted direction of bias due to deviations from intended interventions?		

Domain 2: Risk of bias due to deviations from the intended interventions (effect of adhering to intervention)

Signaling questions	Comments	Response
2.1. Were participants aware of their assigned intervention during the trial?		**NO**
2.2. Were carers and people delivering the interventions aware of participants' assigned intervention during the trial?		**NO**
2.3. [If applicable:] If Y/PY/NI to 2.1 or 2.2: Were important non-protocol interventions balanced across intervention groups?		**NA**
2.4. [If applicable:] Were there failures in implementing the intervention that could have affected the outcome?		**NO**
2.5. [If applicable:] Was there non-adherence to the assigned intervention regimen that could have affected participants' outcomes?	The exercise sessions were well attended (72%) across the exercise programs.	**NO**
2.6. If N/PN/NI to 2.3, or Y/PY/NI to 2.4 or 2.5: Was an appropriate analysis used to estimate the effect of adhering to the intervention?		**NA**
Risk-of-bias judgement		**LOW RISK**
Optional: What is the predicted direction of bias due to deviations from intended interventions?		

Domain 3: Risk of bias due to missing outcome data

Signaling questions	Comments	Response
3.1 Were data for this outcome available for all, or nearly all, participants randomized?	A missing value analysis was performed for all outcome variables.	**YES**
3.2 If N/PN/NI to 3.1: Is there evidence that the result was not biased by missing outcome data?		**NA**
3.3 If N/PN to 3.2: Could missingness in the outcome depend on its true value?		**NA**
3.4 If Y/PY/NI to 3.3: Is it likely that missingness in the outcome depended on its true value?		**NA**
Risk-of-bias judgement		**LOW RISK**
Optional: What is the predicted direction of bias due to missing outcome data?		

Domain 4: Risk of bias in measurement of the outcome

Signaling questions	Comments	Response
4.1 Was the method of measuring the outcome inappropriate?		**NO**
4.2 Could measurement or ascertainment of the outcome have differed between intervention groups?		**Probably NO**
4.3 If N/PN/NI to 4.1 and 4.2: Were outcome assessors aware of the intervention received by study participants?		**No Information**
4.4 If Y/PY/NI to 4.3: Could assessment of the outcome have been influenced by knowledge of intervention received?		**Probably NO**
4.5 If Y/PY/NI to 4.4: Is it likely that assessment of the outcome was influenced by knowledge of intervention received?		**NA**
Risk-of-bias judgement		**LOW RISK**
Optional: What is the predicted direction of bias in measurement of the outcome?		

Domain 5: Risk of bias in selection of the reported result

Signaling questions	Comments	Response
5.1 Were the data that produced this result analyzed in accordance with a pre-specified analysis plan that was finalized before unblinded outcome data were available for analysis?		**YES**
Is the numerical result being assessed likely to have been selected, on the basis of the results, from...		
5.2. ... multiple eligible outcome measurements (eg. scales, definitions, time points) within the outcome domain?		**NO**
5.3 ... multiple eligible analyses of the data?		**NO**
Risk-of-bias judgement		**LOW RISK**
Optional: What is the predicted direction of bias due to selection of the reported result?		

Overall risk of bias: Low risk

26. Keteyian, S. J., Hibner, B. A., Bronsteen, K., et al., (2014). Greater Improvement in Cardiorespiratory Fitness Using Higher-Intensity Interval Training in the Standard Cardiac Rehabilitation Setting. Journal of Cardiopulmonary Rehabilitation and Prevention, 34(2), 98-105. https://doi.org/10.1097/hcr.0000000000000049

Domain 1: Risk of bias arising from the randomization process

Signaling questions	Comments	Response
1.1 Was the allocation sequence random?	Patients were randomized (computerized random number generator transferred to allocation cards and sealed in opaque sequential envelopes) using a 1:1 pattern.	**YES**
1.2 Was the allocation sequence concealed until participants were enrolled and assigned to interventions?		**YES**
1.3 Did baseline differences between intervention groups suggest a problem with the randomization process?	Patient characteristics compared at baseline, using an unpaired t test or the Fisher exact test.	**NO**
Risk-of-bias judgement		**LOW RISK**
Optional: What is the predicted direction of bias arising from the randomization process?		

Domain 2: Risk of bias due to deviations from the intended interventions (effect of assignment to intervention)

Signaling questions	Comments	Response
2.1. Were participants aware of their assigned intervention during the trial?		**NO**
2.2. Were carers and people delivering the interventions aware of participants' assigned intervention during the trial?	The supervisor of the CPX was blinded to study group assignment	**NO**
2.3. If Y/PY/NI to 2.1 or 2.2: Were there deviations from the intended intervention that arose because of the trial context?		**NA**
2.4 If Y/PY to 2.3: Were these deviations likely to have affected the outcome?		**NA**
2.5. If Y/PY/NI to 2.4: Were these deviations from intended intervention balanced between groups?		**NA**

2.6 Was an appropriate analysis used to estimate the effect of assignment to intervention?	To compare between group differences for the changes from baseline to follow up, a student 2-sample t test was used when the assumption of equal variances was not violated; otherwise, a Welch 2-sample t test was used.	**YES**
2.7 If N/PN/NI to 2.6: Was there potential for a substantial impact (on the result) of the failure to analyze participants in the group to which they were randomized?		**NA**
Risk-of-bias judgement		**LOW RISK**
Optional: What is the predicted direction of bias due to deviations from intended interventions?		

Domain 2: Risk of bias due to deviations from the intended interventions (effect of adhering to intervention)

Signaling questions	Comments	Response
2.1. Were participants aware of their assigned intervention during the trial?		**NO**
2.2. Were carers and people delivering the interventions aware of participants' assigned intervention during the trial?		**NO**
2.3. [If applicable:] If Y/PY/NI to 2.1 or 2.2: Were important non-protocol interventions balanced across intervention groups?		**NA**
2.4. [If applicable:] Were there failures in implementing the intervention that could have affected the outcome?		**Probably NO**
2.5. [If applicable:] Was there non-adherence to the assigned intervention regimen that could have affected participants' outcomes?		**Probably NO**
2.6. If N/PN/NI to 2.3, or Y/PY/NI to 2.4 or 2.5: Was an appropriate analysis used to estimate the effect of adhering to the intervention?		**NA**
Risk-of-bias judgement		**LOW RISK**
Optional: What is the predicted direction of bias due to deviations from intended interventions?		

Domain 3: Risk of bias due to missing outcome data

Signaling questions	Comments	Response
3.1 Were data for this outcome available for all, or nearly all, participants randomized?		**YES**
3.2 If N/PN/NI to 3.1: Is there evidence that the result was not biased by missing outcome data?		**NA**
3.3 If N/PN to 3.2: Could missingness in the outcome depend on its true value?		**NA**
3.4 If Y/PY/NI to 3.3: Is it likely that missingness in the outcome depended on its true value?		**NA**
Risk-of-bias judgement		**LOW RISK**
Optional: What is the predicted direction of bias due to missing outcome data?		

Domain 4: Risk of bias in measurement of the outcome

Signaling questions	Comments	Response
4.1 Was the method of measuring the outcome inappropriate?	Symptom-limited CPX tests were performed using a modified-Bruce treadmill protocol.	**NO**
4.2 Could measurement or ascertainment of the outcome have differed between intervention groups?		**Probably NO**
4.3 If N/PN/NI to 4.1 and 4.2: Were outcome assessors aware of the intervention received by study participants?	The supervisor of the CPX was blinded to study group assignment.	**NO**
4.4 If Y/PY/NI to 4.3: Could assessment of the outcome have been influenced by knowledge of intervention received?		**NA**
4.5 If Y/PY/NI to 4.4: Is it likely that assessment of the outcome was influenced by knowledge of intervention received?		**NA**
Risk-of-bias judgement		**LOW RISK**

Optional: What is the predicted direction of bias in measurement of the outcome?		

Domain 5: Risk of bias in selection of the reported result

Signaling questions	Comments	Response
5.1 Were the data that produced this result analyzed in accordance with a pre-specified analysis plan that was finalized before unblinded outcome data were available for analysis?		**YES**
Is the numerical result being assessed likely to have been selected, on the basis of the results, from...		
5.2. ... multiple eligible outcome measurements (eg. scales, definitions, time points) within the outcome domain?		**Probably NO**
5.3 ... multiple eligible analyses of the data?		**Probably NO**
Risk-of-bias judgement		**LOW RISK**
Optional: What is the predicted direction of bias due to selection of the reported result?		

Overall risk of bias: Low risk
